# A Patient with HIV Treated with Ipilimumab and Stereotactic Radiosurgery for Melanoma Metastases to the Brain

**DOI:** 10.1155/2013/946392

**Published:** 2013-12-08

**Authors:** Jacob Ruzevick, Sarah Nicholas, Kristin Redmond, Lawrence Kleinberg, Evan J. Lipson, Michael Lim

**Affiliations:** ^1^Departments of Neurosurgery, The Johns Hopkins University School of Medicine, Phipps 123 600 N. Wolfe Street, Baltimore, MD 21287, USA; ^2^University of Maryland School of Medicine, Baltimore, MD 21210, USA; ^3^Department of Radiation Oncology, The Johns Hopkins University School of Medicine, Baltimore, MD, USA; ^4^Department of Oncology, The Johns Hopkins University School of Medicine, Baltimore, MD, USA

## Abstract

Cancers, such as melanoma, that are associated with immune deficiencies are a major cause of morbidity and mortality in HIV-infected patients. Once patients develop melanoma metastases to the brain, treatment is often limited to palliative surgery and/or radiation. Ipilimumab, a CTLA-4 antagonist, has been shown to improve the median survival of patients with metastatic melanoma. However, available data regarding the safety and efficacy of ipilimumab in HIV-infected patients who develop intracranial melanoma metastases is limited. Here we report our experience administering ipilimumab to a patient with HIV-AIDS who developed multiple intracranial melanoma metastases. Following treatment, our patient showed improvement in systemic tumor control without any apparent interference with antiretroviral treatment.

## 1. Introduction

Melanoma is perhaps the most immunogenic human cancer and, as a result, is more prevalent in HIV-infected individuals than in the general population [1]. Furthermore, HIV-positive patients diagnosed with melanoma experience shorter disease-free survival and overall survival than their immunocompetent counterparts [2]. Currently there is a paucity of data regarding the treatment of patients with HIV who develop intracranial metastases. While surgery and whole brain radiation therapy or stereotactic radiosurgery (SRS) can palliate symptoms of neurologic decline, treatment is almost invariably followed by progression of local and/or systemic disease.

In 2011, the U.S. Food and Drug Administration approved ipilimumab (Yervoy, Bristol-Myers Squibb, Princeton, NJ, USA), a CTLA-4 antagonist, for the treatment of metastatic melanoma after it was shown to improve median overall survival [3]. Recently, ipilimumab has been tested as monotherapy for patients with intracranial melanoma metastases resulting in a median survival of 7 and 14 months in prospective phase II and retrospective studies, respectively [4, 5]. Ipilimumab antagonizes the CTLA-4 protein on the surface of T cells, which acts as coinhibitory signal (i.e., immune checkpoint) after ligand binding. By preventing signaling through the CTLA-4 protein, immune cell tolerance and exhaustion are decreased, leading to a more active antitumor response by the host immune system.

CTLA-4 may also play a role in the progression of HIV. In one study, levels of CTLA-4 expression on CD4+ T cells of HIV-infected patients were inversely correlated with absolute CD4+ T cell count and directly correlated with viral load [6]. Furthermore, CTLA-4 signaling resulted in increased expression of CCR5 and a greater susceptibility of CD4+ cells to infection [7].

The combination of SRS and ipilimumab has recently been studied in a retrospective cohort of patients with melanoma brain metastases and showed improved survival as compared to SRS alone [8]. However, little has been reported about this treatment combination in patients with HIV. Here, we report our experience treating a patient with HIV and multiple brain metastases with ipilimumab and concomitant SRS.

## 2. Case Report

A 48-year-old Caucasian male was diagnosed with HIV-AIDS in 2009 after presenting with oral candidiasis. His absolute CD4 count was 38/mm^3^ with a viral load of 176,000 copies/mL. He began highly active antiretroviral therapy (HAART) with efavirenz/emtricitabine/tenofovir (Atripla, Bristol-Myers Squibb). Also present at the time of presentation was a melanoma on the right cheek, which was subsequently excised. Due to neuropathy, the patient was unable to tolerate lymphoscintigraphy, preventing sentinel node localization.

**Figure 1:**
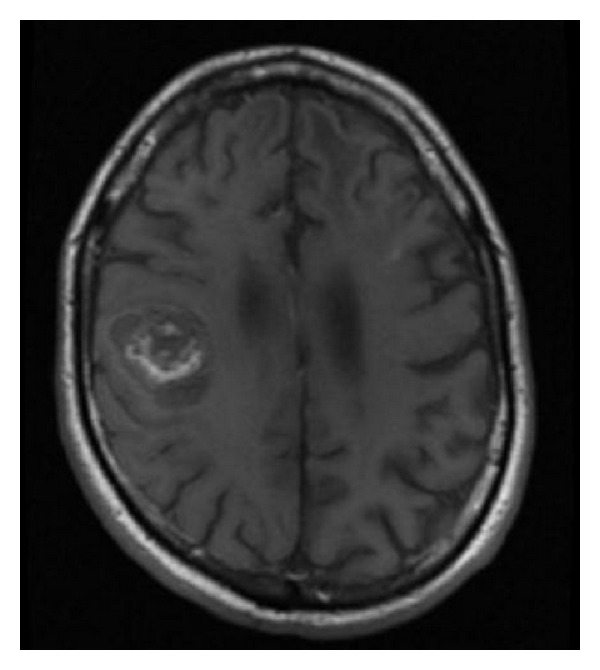
T1-postcontrast MRI showing a 2.7 × 3.5 × 3.1 cm hemorrhagic mass involving the right precentral gyrus. Following surgical resection, pathology confirmed metastatic melanoma.

Two years later, he experienced progressive, left-sided facial, upper extremity, and lower extremity numbness and weakness. MRI of the head and spine revealed a hemorrhagic mass involving the right precentral gyrus measuring 2.7 × 3.5 × 3.1 cm with surrounding vasogenic edema (Figure 1). He was admitted to Johns Hopkins Hospital and underwent a craniotomy for tumor resection. Pathologic evaluation confirmed metastatic melanoma.

Three weeks after surgery, he began treatment with ipilimumab, administered at the standard dose of 3 mg/kg of body weight every three weeks for four doses. One week following the first dose of ipilimumab, he received an SRS boost to the tumor bed of 2100 centigray (cGy) in 3 fractions prescribed to the 73% isodose line using Cyberknife. At the time of the planning MRI, two more brain metastases were noted: one in the right occipital lobe and another in the left frontal lobe, each measuring 7 mm in greatest diameter. Both were treated with SRS 2000 cGy in a single fraction to the 73% isodose line. A restaging FDG-PET/CT scan demonstrated multiple metastases in the right and left hepatic lobes, left iliac external lymph nodes, left deep inguinal nodes, left distal thigh, left popliteal fossa, left anterior tibialis muscle, and right femur.

Eleven days following SRS, he presented to the Emergency Department with weakness in his left upper extremity. An MRI revealed an interval increase in all brain lesions. The right occipital lobe lesion measured 2.4 cm while the left frontal lobe lesion measured 1 cm in greatest diameter. High dose steroids were prescribed as the interval increase in all brain lesions was likely a result of post-SRS inflammation and not true progression of underlying disease.

**Figure 2:**
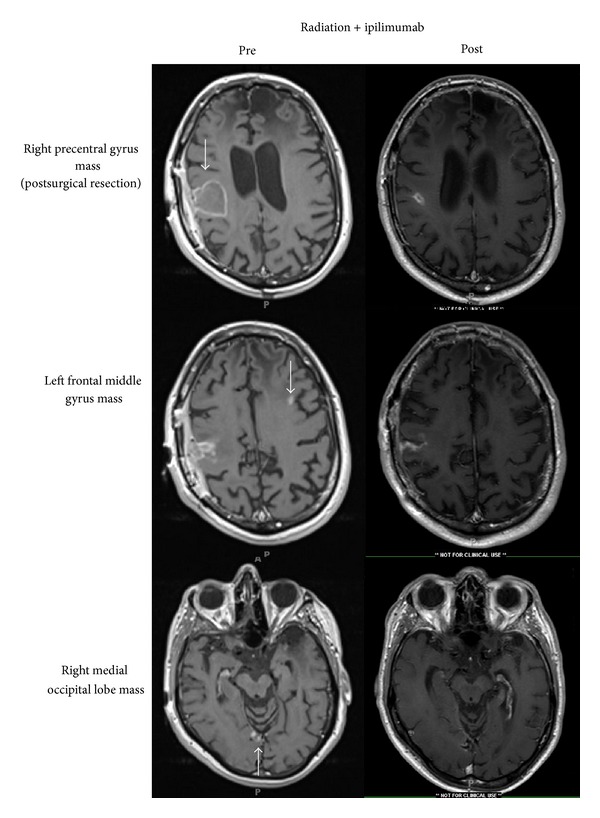
T1-postcontrast MRI showing pretreatment (left column) and 8-month posttreatment (right column) MR imaging of melanoma brain metastases treated with SRS and ipilimumab.

Interval MRI scans over the next 6 months showed stabilization of brain metastases while interval FDG-PET/CT imaging showed stabilization or complete resolution of extracranial lesions. Seven months after completion of SRS, a single new brain metastasis was seen in the left supramarginal gyrus and was treated using SRS 2000 cGy to the 73% isodose line in a single fraction. Representative pretreatment and 8-month posttreatment MR imaging are shown in Figure 2.

At the time of paper preparation the patient is nine months post-surgery after resection of his first brain metastasis. He has remained on efavirenz/emtricitabine/tenofovir, though his adherence to the HAART therapy has been poor. His absolute CD4 counts have ranged from 126 to 449/mm^3^ with an undetectable viral load. He is able to function at his baseline. He experienced only minor gastrointestinal discomfort throughout his treatment with ipilimumab and SRS. His extracranial metastases have remained stable or resolved.

## 3. Discussion

In our patient, the administration of SRS and ipilimumab was well tolerated and caused significant regression of widespread melanoma metastases. Although the benefit of combinatorial therapy in this particular patient is unclear, synergy has been observed in animal models and human trials, suggesting that treatment approaches involving multiple mechanisms of action may be more likely to overcome an immunosuppressive tumor microenvironment than monotherapy [9]. Knisely and colleagues reported in one retrospective study that patients who received ipilimumab in combination with radiotherapy had a median survival of 21.3 months as compared to 4.9 months for patients who received radiotherapy alone [8]. In a prospective, randomized controlled trial, ipilimumab combined with dacarbazine improved overall survival compared to dacarbazine alone in patients with metastatic melanoma [10].

Reports describing HIV-positive patients with metastatic melanoma treated with immunotherapy are limited. Interleukin-2 was tested as an adjuvant to HAART therapy in an attempt to stimulate antitumor activity but did not show a survival benefit in a single patient [11]. Burke and colleagues reported on a single patient with HIV and stage IV melanoma who was treated with ipilimumab and experienced disease regression without dose-limiting toxicities [12].

Preclinical studies describing the role of immune checkpoint molecules, such as CTLA-4, in HIV pathogenesis have also been revealing. One group has reported increased viral replication following blockade of CTLA-4 in primates [13]. Studies of HIV-specific T cells demonstrate expression of programmed death-1 (PD-1), another checkpoint molecule, which is associated with T cell exhaustion and progression of HIV [14]. Blocking the immunoregulatory pathway comprised of either CTLA-4 or PD-1 and its ligands may reinvigorate a host immune response against a chronic infection such as HIV.

Follow-up FDG-PET/CT imaging of our patient after ipilimumab and SRS showed stabilization or regression of multiple systemic metastases. Although the contribution of each therapeutic modality is unknown, a synergy reminiscent of the abscopal effect is possible. The abscopal effect describes a phenomenon wherein a single lesion is irradiated, causing regression of distant metastases. While the exact mechanism remains to be elucidated, the improvement in systemic metastases is likely immune mediated. Lugade et al. reported that melanoma-bearing mice that had been irradiated had improved antigen presentation and an increase in interferon-gamma secreting CD8+ cells after peptide stimulation [15]. Similarly, in a single patient case report, Stamell et al. reported the resolution of systemic melanoma metastases and a greater-than-7-year survival in a patient treated with intracranial radiation in combination with ipilimumab [16].

Based on our patient's experience and the few cases reported in the medical literature, further evaluation of the benefit of radiotherapy and immunotherapies such as ipilimumab to patients with metastatic melanoma and underlying HIV infection is warranted. Outcomes of interest include tumor response to therapy, effect on CD4 count and viral load, and changes in function of HIV-specific T cell function.
